# Performing Illness: A Dialogue About an Invisibly Disabled Dancing Body

**DOI:** 10.3389/fpsyg.2021.566520

**Published:** 2021-05-31

**Authors:** Sarah Pini, Kate Maguire-Rosier

**Affiliations:** ^1^Department of Sports Science and Clinical Biomechanics, University of Southern Denmark, Odense, Denmark; ^2^Department of Theatre and Performance Studies, University of Sydney, Sydney, NSW, Australia

**Keywords:** illness narrative, dance performance, agency, visual autoethnography, disability theory, cancer experience, embodiment, phenomenology of illness

## Introduction

Disability is defined by the World Health Organization as resulting “from the interaction between individuals with a health condition such as cerebral palsy, down syndrome and depression as well as personal and environmental factors including negative attitudes, inaccessible transportation and public buildings, and limited social support” (WHO, 2021). Yet disability scholar Simi Linton notes:

The liberal arts, particularly the humanities, have barely noticed disability beyond the models they accept uncritically, handed down from the sciences and medicine. The tools for inquiry in the humanities have, until recently, rarely been applied to understanding disability as a phenomenon (Linton, 1998, pp. 147–148).

**Figure 1:**
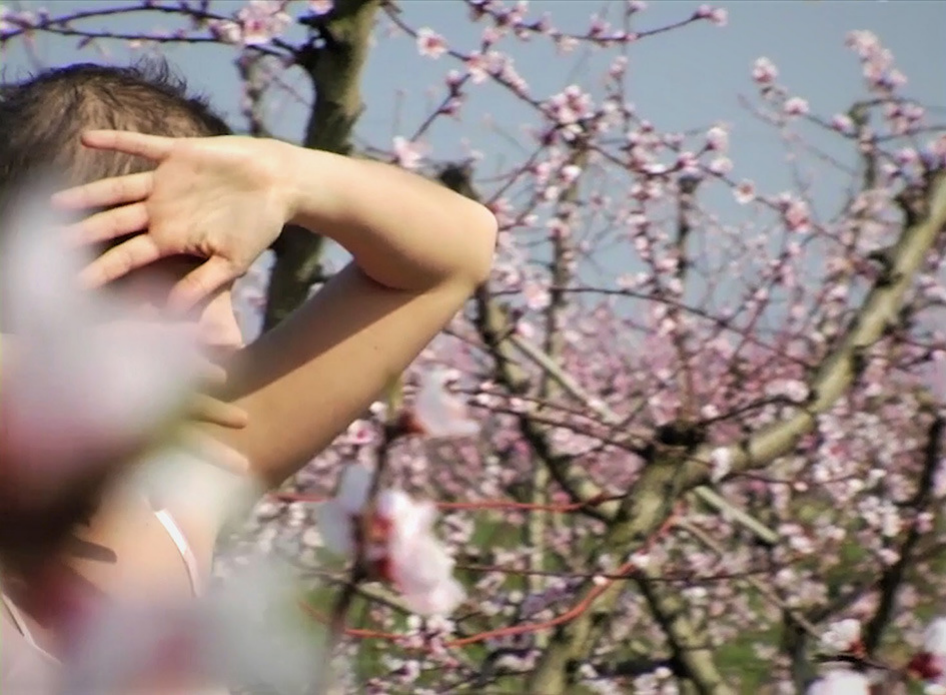
Snapshot from Pini and Pini video article “Resisting the ‘Patient' Body: A Phenomenological Account” (Pini and Pini, 2019).

Global disability communities have famously located disability in the environment. This is recognised as the social model of disability. Social modelling of disability rooted in British activism (Oliver, 1990) diverges from medical modeling—wherein “disability” originates from the individual—by resituating “disability” in the environment. This opinion paper builds from recent efforts in disability studies to define disability experience as intersubjective (Donaldson and Prendergast, 2011; Titchkosky, 2011; Kafer, 2013; Price, 2015) to suggest a move toward an interpretation of disability as an intersubjective ecological phenomenon. To invite this reflection, we discuss the case of Pini's experience of illness and hidden disability related to her oncological treatments for Hodgkin Lymphoma, and the development of her artistic project INFINITO, a longitudinal short dance film series that explores the relationship to cancer and its transformational aspects from a phenomenological and auto-ethnographic perspective (Pini and Pini, 2019) (Figure 1).

This conversational article between two parties—Kate, a disability performance scholar and Sarah, an interdisciplinary artist-scholar with lived experience of disability—considers the dancing body as redeemer in the specific case of experiencing Chemotherapy-Related Cognitive Impairment (CRCI). Through an autoethnographic analysis and phenomenological approach to illness (Carel, 2008, 2016), this article draws on Pini's lived experience of coping with cancer and dancing through illness (Pini and Pini, 2019), in dialogue with Maguire-Rosier's study of dancers with hidden impairments (Gibson and Maguire-Rosier, 2020). This opinion paper is based on an ongoing conversation between the two authors which began at the time of their Ph.D. projects at Macquarie University, Sydney, addressing the dancing body across different but parallel fields: cognitive science (see, for example, Pini et al., 2016; Pini and Sutton, 2021; Pini and Deans, in press) and dance theatre performance (see, for example, Maguire-Rosier, 2016; Czymoch et al., 2020).

In an exploratory account based on an interview with one another, the authors ask: when our senses and perceptions of ourselves and the world that we become are obfuscated, what is the nature of the new relationship between the performing self and its absent body/mind/world?

By focusing on Pini's case, we address the embodied nature of pain and illness, emphasising the role of the body as vehicle of sense making, and how the construction and embodiment of a meaningful self-narrative can help shape one's identity. We relate to notions of agency in psychology and philosophy (Balconi, 2010; Gallagher, 2013; Bresnahan, 2014; Ataria, 2015, 2018; Deans et al., 2015; Moore, 2016; Martens, 2018; Ravn, 2020; Pini and Deans, in press) to elucidate the capacity that dance and creative performance practises exert in enabling the reacquisition of an agentic perspective. Addressing a case of acquired impairment and hidden disability, we explore disability as an intersubjective ecological phenomenon in which the dancing body plays a transformative role. By putting our respective specialisms and embodied experiences in dialogue, our aim with this paper is to offer an interdisciplinary perspective on lived experience of hidden disability in the context of dance.

## Moving in the Fog

**Kate:** Our discussion departs from critiques of social constructivism that highlight how the social model of disability reinstates body–mind dualisms (Shakespeare, 2006). As disability scholar Hughes (Hughes, 2009, pp. 399–401) explains, the body itself is social, but the inverse is also true, that is, the social is bodily. Therefore, rather than seeing the “body” as a “sociopolitically constituted and material entity” produced by both “disability” and “impairment” (Price, 2015), we echo Price's concept of the “bodymind” in disability studies (Price, 2015, p. 271), to move toward an embodied understanding of disability. In this, the Cartesian body–mind split is negated, nodding toward Merleau-Ponty's theory of embodied perception (Merleau-Ponty, 2013[1962]), and accounting for the possibility of mental or cognitive disability and gesturing toward disability as, indeed, an intersubjective event. How did your experience of illness and its implications impact you, Sarah?

**Sarah:** During my 10-year long journey with cancer, I experienced “chemo fog,” in medicine known as Chemotherapy-Related Cognitive Impairment (CRCI), a little-known condition that goes by the lay term of “chemobrain” or “chemo fog” (Williams et al., 2016). Chemo fog refers to a collection of deficits in memory, attention, concentration, and executive function that affect patients who undergo chemotherapy treatments. Typical symptoms of chemobrain include forgetfulness, impaired concentration and attention, difficulty with multitasking and with word recall, short-term memory loss, and often the inability to organise daily tasks (Asher, 2011; Asher and Myers, 2015). Silverman and Davidson provide several accounts from cancer patients who have experienced chemobrain. To emphasise the sense of frustration and psychological pain tied to such experience, they report a quote from Jackson Hunsicker, an American writer, TV, and film director, who said: “It is painful when people look at me with confusion while I am trying to talk. I know that I'm not making sense, and I don't know how else to talk. When it happens, I die a million deaths and feel very dumb” (Silverman and Davidson, 2009, p. 47). As Silverman and Davidson stress, chemotherapy treatments not only disrupt brain function, but also have a deep impact on the psychological well-being of those affected. Such cognitive impairments deeply influence the social life and interpersonal exchanges of these patients, contributing to a rising sense of alienation and frustration and in some cases a deep sense of self-doubt (Silverman and Davidson, 2009). I went through a similar experience after I underwent my allogeneic hematopoietic stem cell transplantation (Allo-HSCT) in 2015, when a feeling of estrangement and disconnection from myself and the world reached an unprecedented level of intensity. Given the high toxicity of the conditioning chemotherapeutic regimen employed with this procedure, several clinical studies have investigated CRCI in haematological patients undergoing this type of transplant (Syrjala et al., 2011; Scherwath et al., 2013; Sharafeldin et al., 2017). Such conditioning regimens and related toxicities alter brain metabolism, resulting in a long-lasting decrease in cognitive function, which is a common complication for patients undergoing HSCT (Maffini et al., 2017). Neurocognitive dysfunctions range from subtle to severe and can last years after treatment (Williams et al., 2016; Kelly et al., 2018).

For a couple of years after my transplant I struggled with focusing on the task at hand; even performing what had previously been simple habitual activities became a difficult job. It was not only verbal retrieval and the ability to retain information in short-term memory that were impaired, but also my motor control and manual dexterity. During the years following my transplant, I was constantly forgetting things, dropping objects, and missing information. I could not follow a discussion if it involved listening and responding to more than one person, and simply retaining basic information became a real challenge. I was feeling depressed, terribly clumsy, and isolated. My relationship to the world thus felt syncopated, unsynchronised, and (somewhat paradoxically) constantly lagging behind.

During this time, feeling my own presence “in-the-world” became an impossible task as I was experiencing the world at an increased distance, as if it was hurtling away from me, spinning around me at a pace I could no longer keep.

## Dancing Illness

**Kate:** How do you shape your life narrative and articulate who you are, what you are doing or where you are going, when you are moving in the “fog?”

**Sarah:** The intense experience of “losing” presence to the chemo-fog made me realise the central role played by the body in the construction of sense-making. Illness narratives and the concept of emplotment (Mattingly, 1994)—a series of biographical events constructed retrospectively into a narrative—play crucial roles in the rehabilitation of people experiencing illness and attendant trauma. Carel considers the illness experience a “violent” invitation to philosophise and to find reasons to carry out a meaningful and happy life despite the limitations imposed by illness (Carel, 2008). In my experience the illness event became a “violent” invitation to dance. Throughout my 10-years long experience of illness, I developed an artistic embodied practice by performing, recording, and collecting video dance performances enacted either during, before or after my medical treatments.

Later on, I began “distilling” these performances into distinct visual experiments, clustered together under the project INFINITO, a longitudinal short dance film series that explores the relationship to illness and its transformational aspects from a phenomenological and auto-ethnographic perspective. The first episode of the series, the short film ABISSO (Pini and Pini, 2018) captures this feeling of disconnection, of being immersed in a different dimension, separate from the rest of the world, a dimension where it is impossible to breathe^1^.

Depending on the specific situation I was finding myself in during my medical journey, and the type of challenge I was facing (such as beginning a new treatment after the failure of previous protocols or coping with the disruptive side effects of chemotherapy affecting my identity and sense of self), these dance performances allowed me to express and modulate the extent of psychological, emotional, physical, and social pain I was going through, allowing me to regain an agentic perspective.

Several interdisciplinary approaches across philosophy and the cognitive sciences have addressed the concept of the sense of agency, pointing out its inherent complexity, and phenomenological ambiguity (Gallagher, 2013). To simplify, a common definition of agency in psychology contends the feeling of control over someone's own bodily actions (Balconi, 2010; Moore, 2016)^2^. In the context of improvisational dance practises, agency is understood as “the control and intention the dance performer has to move in a certain way” (Bresnahan, 2014, p. 86). From a phenomenological enactive perspective, sense of agency is understood as a “heterogenous collection of different ways or aspects of feeling in control that depends on context, the task, and the person's history and capacities” (Buhrmann and Di Paolo, 2017, p. 228). Research across phenomenology and the cognitive sciences pointed out how undergoing traumatic experiences often leads to a diminished sense of agency and a feeling of dissociation and “disownership” (Ataria, 2015, 2018). Researchers in psychology have also argued that a sense of agency arises from the coupling of the self with the social and material world, and as such it presents an interpersonal origin (Deans et al., 2015)^3^. Understanding agency and attunement as an intersubjective ecological phenomenon is salient in the context of addressing performance practises and the ways dance can expand and influence empathic and perceptive experience (Pini and Deans, in press). As I have discussed in my video-essay, “the practice of performing my illness helped the construction of sense-making and sustained the process of elaboration of my own narrative” (Pini and Pini, 2019, p. 8).

What guided my inquiry was the desire to discover how I could regain an agentic perspective “in-the-world” and re-entering the social life that was left behind. The possibility of reshaping my relation to the world, to re-experience presence through the body, would enable me to dive back into what Carel calls the “flowing river” of conscious experience (Carel, 2008, p. 123). These dance performances allowed me to re-enact my way toward-the-world, providing the chance to “shape my identity in other and more endurable terms than the ones provided by the biomedical model and cancer's common rhetoric” (Pini and Pini, 2019, p. 8). By engaging with embodied narrative and my dance practise I could not only recover my “absent” presence but also find a renewed version of what Gumbrecht calls “the feeling of *being in sync with the things of the world*” (Gumbrecht, 2004, p. 117—emphasis in original).

**Kate:** In the context of dance by artists with disability, issues of pain and vulnerability remain largely unexplored. So for me, your artistic enquiry, Sarah, is unique^4^. What I find interesting is that the concept of pain undermines popular social constructivist approaches to disability. These approaches disavow, in Anna Hickey-Moody's words, “the viscerally intense, complex and laborious nature of the lives of people with disabilities” (Hickey-Moody, 2010, p. 509). For artists with hidden disability, then, this means their links to the political movement heralding the social model of disability, on which the disability arts sector is largely founded (Hadley, 2017; Hadley and McDonald, 2019), are somewhat severed. Is—or was—this the case for you, Sarah?

**Sarah:** I think I can relate to that. In my own experience I often found the common rhetoric of cancer somewhat negates the possibility of fully embracing an agentic perspective. In my view this is due to the predominant role that the war-like metaphor plays in this discourse. The illness is often described as “other,” as the “enemy”—which of course is something undesirable we want to get rid of—but without quite considering that the person affected by this illness has to “go into battle,” waging war to cancer, by engaging into an unfairly difficult war since the battlefield is her own body. In my opinion, what is most needed, rather than a warfare rhetoric, would be a deeper understanding of the situation the patient is going through, that inevitably includes the embodied nature of pain and illness, and the central role that the body plays in shaping cognitive experience. I have often wondered what is at stake when these hidden experiences remain unexplored and underrepresented in cultural texts, how can the painful experience be recognised in the first place?

## Seeing and Sensing Hidden Disability

**Kate:** At the time, these markers of disability that you recount, Sarah, would have made visible your otherwise hidden experience of illness. To my mind, this resonates with the recent “emotional turn” in disability studies. According to Donaldson and Prendergast, the idea of emotion itself is relatively new in disability theory; “since Shapiro's *No Pity* in 1993, there is definitely no crying in disability studies” (Donaldson and Prendergast, 2011, p. 129). Peripheral to the celebratory agenda of mainstream disability studies, Kafer (2016) calls for the formation of disability theories of trauma, loss and mourning as valid cultural sites within which to explore the concept of disability. Feminist disability scholarship has critiqued the silence of experiences of pain and vulnerability in social model accounts of disability. According to Price:

The problem is one of judgement: We wish to celebrate difference, or at least to avoid saying that one manifestation of personhood (being disabled) is worse than any other. Yet, at the same time, merely by positing desires, we *a priori* cannot help mapping the undesirable (Price, 2015, p. 276, original emphasis).

Through a disability lens, the predicament of pain is precisely that it—like vulnerability and shame—is undesirable. This inescapability of the undesirable can only be resolved if disability desires its undesirable subject—pain. Price stresses the importance of responding to another's experience of pain as “real,” present and necessary. Similarly, Dokumaci's call to “accommodate pain” (Dokumaci, 2013, p. 112) when interacting with one's environment treats pain as something to embrace, not reject.

## Discussion

We focus on Pini's re-enactment of illness through her video dance performances, to emphasise how performative practises can offer original perspectives that contribute to questioning and reframing established notions of subjectivity and different forms of agency. By relating to current notions of agency in psychology and philosophy (Balconi, 2010; Gallagher, 2013; Bresnahan, 2014; Ataria, 2015, 2018; Deans et al., 2015; Moore, 2016; Martens, 2018; Ravn, 2020; Pini and Deans, in press), we stressed the capacity of dance to enable the reacquisition of an agentic perspective. Addressing Pini's case of acquired impairment and hidden disability, we highlighted how agency in dance encompasses cognitive and performative dimensions, providing an example of how a dancing body can reframe notions of disability considering its intersubjective and ecological aspects.

Artists and scholars are treading a fine line between staging a personal storey of pain laid bare in all its intelligence, wonder, and “realness,” and avoiding perpetuating the medical model of disability which holds hostage the narrative of disability as one of pride, gain, and so on. An antidote to the complexity of painful experience is recognising it, like any experience, as intersubjective. As performance artist and disability scholar Crow perceives, “impairment is the functional limitation(s) which affect a person's body, [while] disability is the loss or limitation of opportunities resulting from direct and indirect discrimination” (Crow, 1996, p. 208). As such, the environment either enables or disables agency, and produces disability or not.

In disability studies there have been recent efforts to define disability experience as intersubjective. For example, Titchkosky (2011), Donaldson and Prendergast (2011), Kafer (2013), and Price (2015) adopt a relational perspective to approach disability ontology. Even though performance artist and disability activist Crow finds social modelling of disability liberating, she nonetheless expresses that it “present[s] impairment as irrelevant, neutral and, sometimes, positive, but never, ever as the quandary it really is” (Crow, 1996, p. 208). Moreover, this quandary is notoriously regarded as “suspect” by onlookers who don't “see” a disability (Cumings, 2016, p. 153; see also Montgomery, 2001). So, we use the term “hidden” in light of Montgomery's critique of “invisible disability” where the focus is on disability or impairment conceived as visual (Montgomery, 2001). Kafer tellingly yields, “I am not interested in becoming more disabled than I already am” (Kafer, 2013, p. 4). The lived experience of pain, emotion, and other hidden impairments is however, not limited to the impairments themselves, but rather extends to the world. As Thomas adds, there is a key difference between what she calls “impairment” and “impairment effects”: she states, “the fact that I cannot hold a spoon or saucepan in my left hand is an effect of my impairment and does not constitute disability” (Thomas, 1999, p. 43). The lack of cultural awareness of such experiences means hidden impairments are frequently misunderstood and devalued within the disability community. In social spaces where these lived experiences fail to be perceived, very different issues can arise.

With this opinion paper we do not mean to suggest hidden disability experiences are—or feel—deficit. Rather, we suggest that the experience of hidden impairment can be deeply emotional precisely because it is not discernible to others. As Pini's experience suggests—in the case of a professional dancer, and thus, a highly trained performing body-mind—we imagine exploring this state. Where confusion, reverberating trauma and thinking itself fuses one's world into a perpetual condition of uncertainty or frustration, a deep intelligence and sense of curiosity are enabled.

## Conclusion

Pini's visual auto-ethnographic account illustrates how dance and performative practises can offer ground for transformation and reshape an agentic perspective over a disrupted life narrative, viscerally perceived in her filmic essay (Pini and Pini, 2019). Whereas, Pini's dancing body recovered an agentic perspective in her experience of alienation and frustration tied to chemo fog and related impairments, this same dancing body now—later—resists theoretical diagnosis. While our discussion reveals that Pini's dancing body elucidates healing, it beckons more than therapy, perhaps even more than art. In the emergence of somewhat “enminded” movement, to borrow Kuppers' corrupted derivative of “embodiment” (Kuppers, 2014, pp. 43–44), we suggest Pini's physical exploration of her ever-changing chimaera-like form catalysed an ultimately emancipatory process. By drawing from Pini's lived experience of hidden impairment in relation to her artistic and scholarly work, we framed a lived experience of disability as interrelational, recognising the transformative potential of performing pain and dancing through illness. With this dialogue we emphasise the role played by dance and embodied performative practises in contributing to fostering a sense of agency in the face of illness and disruptive events, indicating parallels between psychology and dance studies. Rather than being an exhaustive argument, this dialogical paper seeks to capture possibilities of future directions into interdisciplinary research transcending cognitive sciences and the arts and humanities.

## Ethics Statement

Written informed consent was obtained from the individual for the publication of any potentially identifiable images or data included in this article.

## Author Contributions

All authors listed have made a substantial, direct and intellectual contribution to the work, and approved it for publication.

## Conflict of Interest

The authors declare that the research was conducted in the absence of any commercial or financial relationships that could be construed as a potential conflict of interest.

## Footnotes

^1^The short film ABISSO (2018) can be watched at the following link https://vimeo.com/user17123701. ABISSO has been selected for international film festivals including Dance Cinema, [C]Screen-Spring Dancefilm Festival 2020, Moving Images International videodance festival 2020, IntimateLens 2019, among others. ABISSO received several acknowledgments including the 2019 CHASS Australia Prize for a Student in the Humanities, Arts, and Social Sciences, and won the 2018 Visual and Creative Ethnography Competition organised by the Australian Anthropological Society (AAS) and Australian Network of Student Anthropologists (ANSA). For further information on the creative process underling the development of the film ABISSO, see the interview to Sarah Pini published on Dance Cinema.org https://www.dancecinema.org/abisso.html.

^2^For an overview of the relationship between sense of agency and consciousness across interdisciplinary approaches see Obhi et al. (2015).

^3^For further elucidations on the difference between “sense of other” and “sense of us” and collective agency see Martens (2018).

^4^For insights into screendance and disability on screen see Whatley (2010).
